# The Effect of Early Spironolactone Administration on 2-Year Acute Graft Rejection in Cardiac Transplant Patients

**DOI:** 10.3390/biomedicines13051164

**Published:** 2025-05-10

**Authors:** Dragos-Florin Baba, Alina Danilesco, Horatiu Suciu, Calin Avram, Marius Mihai Harpa, Mircea Stoian, Diana-Andreea Moldovan, Laurentiu Huma, Gabriel Rusu, Tunde Pal, Adina Stoian, Anca-Ileana Sin

**Affiliations:** 1 Department of Cell and Molecular Biology, George Emil Palade University of Medicine, Pharmacy, Science and Technology of Targu Mures, 540142 Targu Mures, Romania; dragos-florin.baba@umfst.ro (D.-F.B.); ileana.sin@umfst.ro (A.-I.S.); 2 Emergency Institute for Cardiovascular Diseases and Transplant, 540136 Targu Mures, Romania; horatiu.suciu@umfst.ro (H.S.); mariusharpa@gmail.com (M.M.H.); diana.moldovan@umfst.ro (D.-A.M.); gabriel.rusu@ibcvt.ro (G.R.); 3 Targu-Mures County Hospital, 540072 Targu Mures, Romania; alinka942@yahoo.com; 4 Department of Surgery, George Emil Palade University of Medicine, Pharmacy, Science and Technology of Targu Mures, 540142 Targu Mures, Romania; 5 Department of Medical Informatics and Biostatics, George Emil Palade University of Medicine, Pharmacy, Science and Technology of Targu Mures, 540142 Targu Mures, Romania; 6 Department of Anesthesiology and Intensive Care, George Emil Palade University of Medicine, Pharmacy, Science and Technology of Targu Mures, 540139 Targu Mures, Romania; mircea.stoian@umfst.ro; 7 Department of Family Medicine, George Emil Palade University of Medicine, Pharmacy, Science and Technology of Targu Mures, 540142 Targu Mures, Romania; 8 Department of Internal Medicine V, George Emil Palade University of Medicine, Pharmacy, Science and Technology of Targu Mures, 540136 Targu Mures, Romania; tunde.pal@umfst.ro; 9 Department of Pathophysiology, George Emil Palade University of Medicine, Pharmacy, Science and Technology of Targu Mures, 540136 Targu Mures, Romania; adina.stoian@umfst.ro

**Keywords:** cardiac transplantation, mineralocorticoid receptor antagonist (MRA), spironolactone, acute graft rejection (AGR), mortality

## Abstract

**Background:** The objective of our study was to investigate the impact of mineralocorticoid receptor antagonists (MRAs), such as spironolactone, administrated early after cardiac transplantation on the occurrence of acute graft rejection (AGR) in the first 2 years post-transplant. **Methods:** This retrospective research was conducted in the Emergency Institute for Cardiovascular Diseases and Transplantation of Targu Mures, Romania. After applying the inclusion criteria, between January 2011 and December 2023, 36 patients fit the study design. Using Cox proportional hazards regression and Kaplan–Meier curves, we determined the time-to-event distribution, for which the first episode of AGR was considered an event, with a significance threshold of 0.05. **Results:** The 1-year rate of AGR was 38.9% and was 47.2% at 2 years, with a 2-year mortality of 11.1%. The interpretation of the Cox regression indicated that early initiation of spironolactone represents a protective factor against the 2-year AGR (HR: 0.263; 95%CI: 0.076–0.922; *p* = 0.037 by the log-rank test). **Conclusions:** These results might suggest a possible benefit of the early administration of spironolactone after a heart transplant, but further prospective studies need to be performed for the validation of our findings.

## 1. Introduction

Heart transplantation represents a well-established therapy for patients diagnosed with end-stage heart failure (HF), in which all therapeutic alternatives have been exhausted [1,2]. In the past few years, there has been progress in the survival and quality of life of heart transplant patients [3]. However, contemporary research tends to focus on immunosuppressive therapy, with the purpose of overcoming the possible short-term complications associated with the postoperative treatment. On the opposite side of the indisputable benefits brought by immunosuppressive treatment to the overall survival of heart transplant patients are the possible complications, most notably infections and acute graft rejection (AGR) [4]. Younger patients seem to frequently suffer fatal episodes of AGR, in contrast with the elderly, who have chronic graft rejection and primary graft dysfunction as the main causes of death [5].

Allograft rejection can be divided into three types: hyperacute, acute, and chronic rejection [6]. AGR, as the most common type, is responsible for approximately 10% of deaths in the first three years post-transplantation and is primarily mediated by T cells, along with macrophages and lymphocytes [6,7,8]. Secondarily, it can be antibody-mediated, in which case the antigen–antibody reaction between the donor HLA system and recipient antibodies leads to capillary endothelial damage [9]. In cellular rejection, there are several mechanisms involved, such as complement activation, direct lysis of graft cells by T cells, and alloimmunity induced by major and minor histocompatibility antigens [10].

The early induction of postoperative immunosuppressive treatment can be achieved through rabbit antithymocyte globulin (R-ATG), with reasonable rates of complications. Nonetheless, the administration of these antibodies requires caution because of the paucity of recommendations of the International Society for Heart and Lung Transplantation (ISHLT) guidelines [11,12]. A recent meta-analysis, including 7 randomized controlled trials and 12 observational studies, showed possible superiority of R-ATG compared to other induction agents, such as basiliximab [13]. There is evidence that the administration of R-ATG early after graft transplantation represents a cost-effective solution in reducing the risk of AGR, furthermore increasing long-term survival [14,15]. On the other hand, in lung transplant patients, a randomized controlled trial showed that induction therapy with daclizumab, an interleukin-2 receptor blocker, reduced the incidence of AGR compared with R-ATG [16]. However, R-ATG seems to increase the risk of wound complications, postoperative acute kidney injury, and cardiovascular dysfunction [17,18].

Traditional maintenance therapy after a heart transplant includes corticosteroids, cyclosporine or tacrolimus, and azathioprine or mycophenolate mofetil. Recently, new agents were tested in order to reduce the usage of calcineurin inhibitors and corticosteroids in order to decrease the rate of late complications [19]. In terms of the possible maintenance therapy combinations, Kobashigawa et al. compared the administrations of steroids associated with either tacrolimus with sirolimus, tacrolimus with mycophenolate mofetil, or cyclosporine with mycophenolate mofetil. In cardiac transplant patients, the combination of tacrolimus with sirolimus or cyclosporine with mycophenolate mofetil reduced the risk of high-grade AGR at 1 year, also improving the side-effect profile [20].

With the exception of immunosuppressive treatment, there is a lack of data on the benefits of other drug therapies on the management of heart transplant patients. BBs appear to be efficient in heart transplant patients who suffer from sinus tachycardia, supraventricular and ventricular tachyarrhythmias, left ventricular systolic dysfunction, and arterial hypertension [21]. There is evidence that increased heart rate after heart transplant represents an independent predictor of all-cause mortality [22], suggesting a potential mechanism through which BBs may reduce mortality in this category of patients.

The administration of angiotensin-converting enzyme inhibitors (ACEis) has been proven to be a reliable therapeutic option for arterial hypertension on account of the widespread molecular effects that also impact heart transplant receipts [23]. In 2020, a prospective, randomized trial that tested the impact of early initiation of ramipril after heart transplant showed that ACEi may have favorable long-term clinical outcomes, effectively lowering blood pressure, with no negative impact on the patients’ safety [24].

A meta-analysis that investigated the role of statins after cardiac transplant highlighted lower mortality rates in patients who benefit from this drug treatment, decreasing the rates of fatal rejection episodes, terminal cancer risk, and coronary vasculopathy incidence [25,26,27]. In one of our studies, preoperative statin administration was associated with a lower risk of early postoperative outcomes, but with a higher risk of newly diagnosed type 2 diabetes mellitus (T2DM) and acute kidney injury [28].

The impact of spironolactone administration in the prognosis of heart transplant patients is one of the less investigated areas in the current literature. The objective of this retrospective study was to determine the relationship between early spironolactone administration after cardiac transplantation and the incidence of AGR in the first 2 years post-transplant.

## 2. Materials and Methods

**Figure 1:**
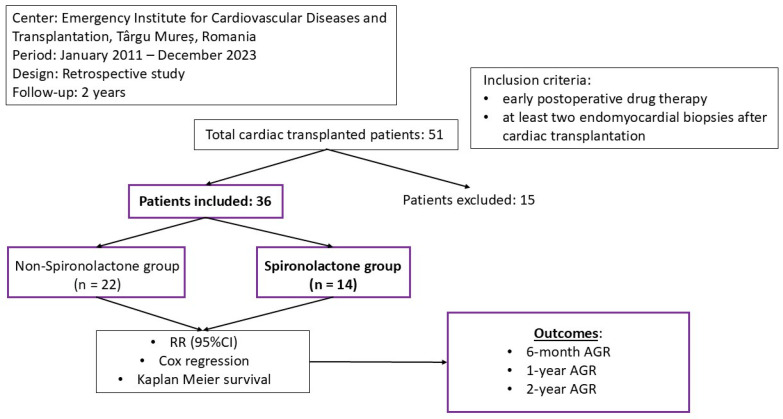
The design of our study.

Our study was conducted under the form of retrospective research in the Emergency Institute for Cardiovascular Diseases and Transplantation of Targu Mureș, Romania. Between January 2011 and December 2023, a total of 51 patients underwent cardiac transplantation. As inclusion criteria, we listed the presence of at least two endomyocardial biopsies over a 2-year period and a detailed report of the postoperative drug treatment. After selection, 36 patients fit the study design. The research was conducted according to the Declaration of Helsinki. The protocol validation was obtained by the ethics committee of the Cardiovascular and Transplantation Emergency Institute of Targu Mureș. The endomyocardial biopsies were performed in the first 3 months after heart transplantation, with further biopsies on a yearly based protocol. The 6-month survival was determined through interrogation of the register available in the intranet system of the Emergency Institute for Cardiovascular Diseases and Transplantation of Targu Mureș (Figure 1).

Early initiation of spironolactone was considered up to 10 days after heart transplant. Firstly, the cohort was distributed in two different groups based on the administration of spironolactone. The baseline characteristics of patients included the daily dosage of spironolactone, age, gender, body mass index (BMI), body surface area (BSA), ischemic etiology of the cardiomyopathy (CM), previous diagnosis of T2DM, chronic prior treatment with BBs, carvedilol, ACEIs, ramipril, MRAs, spironolactone, duration of inotropes/vasopressors, intensive care unit (ICU) stay, hospitalization duration, and 6-month and 2-year mortality rate. The survival rate of the cohort was obtained by using the software of our institute.

The statistical analysis was performed using Stata software version 18.0 (StataCorp, College Station, TX, USA), determining the mean values, standard deviation (SD), medians, 25th–75th interquartile rang (IQR), and the rate of events in the spironolactone and non-spironolactone groups. The normality test was evaluated by the Shapiro–Wilk test [29]. We compared the values between the spironolactone group and non-spironolactone group using Student’s *t* test for parametric data, the Mann–Whitney test for non-parametric data, and the Chi-square test for categorical data. We estimated the relative risk (RR), corresponding 95 percent confidence interval (95%CI), and the number needed to treat (NNT) between the spironolactone treatment and outcomes (6-month AGR, 1-year AGR, and 2-year AGR). Moreover, the average treatment effect was assessed by inverse probability weighting (IPTW). Using Cox regression with the Breslow method for ties and Kaplan–Meier curves, we determined the time-to-event distribution, the first episode of AGR being noted as an event. The presence of acute cellular rejection (ACR) or antibody-mediated rejection (AMR) were recognized as AGR. The significance threshold was set at 0.05.

## 3. Results

From the total number of 36 patients included, 14 had early postoperative treatment with spironolactone. The mean age in the cohort was 40.417 years (SD = 13.561 years), including three females (8.3%), the mean BMI was 23.439 kg/m^2^ (SD = 4.574 kg/m^2^), and the mean BSA was 1.849 m^2^ (SD = 0.319 m^2^). The oldest patient from our cohort was 61 years old, and the youngest was 11. Ischemic CM before heart transplantation was seen in eight patients (22.2%), and three individuals had T2DM (8.3%). A total of 28 patients (77.8%) had previous chronic treatment with BBs, out of which 25 were treated with carvedilol (69.4%), and 17 had previous treatment with ACEIs (47.2%), out of which 12 were treated with ramipril (33.3%); 33 patients had previous treatment with MRAs (91.7%), with 29 having prior therapy with spironolactone (80.6%). The mean usage of postoperative inotropes/vasopressors was 6.806 days (SD = 6.765 days), and a mean ICU stay of 53.056 days (SD = 68.201 days) was recorded. The mean duration of hospitalization was 61.833 days (SD = 71.952 days), with a 2-year mortality rate of 11.1% (4:36) (Table 1).

Looking at the AGR, at 6 months, 13 patients (36.1%) experienced the first episode of AGR; 14 (38.9%) experienced this at 1 year and 17 patients at 2 years (47.2%), with only 3 of the patients experiencing moderate/severe AGR (8.3%). The 6-month AGR rate of the spironolactone group was 14.3% (2 out of 14 patients) compared with 50.0% in the non-spironolactone group (11 out of 22 patients). The 6-month and 2-year mortality rate was 7.1% in spironolactone group compared with 13.6% in non-spironolactone group (Table 1).

At 1 year, 14.3% (2 out of 14) of patients had experienced AGR in the spironolactone group compared to 54.5% (12 out of 22 patients) in the non-spironolactone group. At 2 years, 21.4% of patients had AGR in the spironolactone group (3 out of 14 patients) versus 63.6% in the non-spironolactone group (14 out of 22 patients).

**Figure 2:**
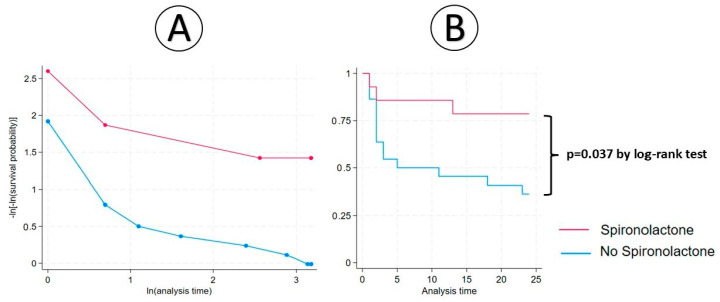
Cox regression (**A**) and Kaplan–Meier analysis (**B**) between spironolactone therapy and 2-year AGR.

No significant association was determined in terms of the 6-month AGR (RR = 0.286; 95%CI: 0.074–1.10;1 *p* = 0.068). The early initiation of spironolactone after cardiac transplantation represented a protective factor against 1-year AGR (RR = 0.262; 95%CI: 0.069–0.999; *p* = 0.049) and 2-year AGR (RR = 0.337; 95%CI: 0.118–0.964; *p* = 0.042) (Table 2). Using the IPW, there were significant differences between groups in 6-month AGR (*p* = 0.012), 1-year AGR (*p* = 0.004), and in 2 year-AGR (*p* = 0.005). By Cox regression and Kaplan–Meier survival, we observed that the 2-year AGR hazard ratio of the spironolactone group was 0.263 (95%CI: 0.076–0.922; *p* = 0.037 by the log-rank test) (Figure 2).

In terms of immunosuppressive therapy, all patients received induction therapy with R-ATG and intravenous methylprednisolone. Afterwards, all survivors had triple maintenance treatment with tacrolimus, mycophenolate mofetil, and prednisone. The oldest patient who experienced 2-year AGR was 54 years old at the time of transplant, while the youngest was 11 years old, both receiving spironolactone in the early postoperative stages. Of all the patients with AGR in the first 2 years post-transplant, eight had ACR (47.1%) and nine AMR (52.9%) (Table 3).

From the group with early spironolactone initiation, two out of three patients (66.6%) experienced mild AGR. Two of these rejections were ACR, and one AMR. In patients without post-transplant spironolactone administration, 12 out of 14 patients (85.7%) experienced mild AGR. One patient had moderate AMR (grade 2), and a single individual experienced severe AGR (grade 3), with prolonged ICU stay and hospitalization (Table 3).

## 4. Discussion

Post-transplant immunosuppression and its constant development have considerably reduced the frequency of graft rejection. Among available medications, such as corticosteroids, antimetabolites (azathioprine and mycophenolat mofetil), and antiproliferative drugs (sirolimus and cyclosporine), modern immunosuppressive therapy can include, in particular cases, monoclonal antibodies and extracorporeal photoimmune therapy [30,31,32]. In our study, the 2-year rate of AGR was 47.2%. Early spironolactone initiation, at a mean dosage of 33.3 mg, seemed to be a protective factor against 1-year and 2-year AGR, with no significant influence at the 6-month threshold. The rates of AGR in patients with spironolactone therapy were lower than the ones of individuals who did not receive the drug. This relation was observable at 6 months (14.3% versus 50%), 1 year (14.3% versus 54.5%), and 2 years (21.4% versus 63.6%). Moreover, there is a possible benefit of spironolactone administration in the patients’ survival rate at 6 months and 2 years after heart transplant.

The role of aldosterone in organ transplantation was previously studied. Aldosterone is the primary mineralocorticoid that regulates homeostatic volumes and the concentration of blood electrolytes. However, excessive levels of this hormone were associated with arterial hypertension and major cardiovascular complications, such as coronary artery disease or stroke. It has been proven that this is a consequence of inflammation, which contributes to vascular remodeling, fibrosis, and endothelial dysfunction and increases reactive oxygen species (ROS) levels [33]. The proinflammatory status before heart surgery seems to be associated with worse postoperative outcomes, a relationship which is present very early after cardiac transplantation [34,35,36].

T lymphocytes play an important role in AGR [10]. By stimulating dendritic cells, aldosterone activates CD8+ T cells and polarizes CD4+ T cells to Th17, a phenotype associated with inflammation and autoimmune disease, secondary to the production of interleukin-6 (IL-6) and tumor necrosis factor-α (TNF-α) [37]. There is evidence that spironolactone possesses immunomodulatory effects, inhibiting the activation of the MRs on the surface of T cells, influencing the T lymphocyte and IL-17 pathways [38].

The main cause of allograft rejection has been attributed to macrophage accumulation [39]. Type 1 macrophages (M1) are considered to be proinflammatory cells with the role of secreting proinflammatory cytokines, such as IL-1, IL-6, TNF-α, and IL-23 [40]. Experimental and pre-clinical studies have shown that IL-6 inhibition may be successful in preventing acute and chronic cardiac allograft rejection [41]. An experimental model highlighted that aldosterone, in the presence of sodium, increased M1 macrophage infiltration, an effect which was attenuated by spironolactone administration [42].

MRs are also expressed in fibroblasts, contributing to inflammatory macrophage polarization, myofibroblast differentiation, and progressive fibrosis [43]. It has been noted that cardiac fibrosis, due to chronic allograft rejection, is mainly a consequence of the activity of intracardiac fibroblasts [44]. Furthermore, proinflammatory cytokines (IL-6) induce collagen synthesis in fibroblasts and shift their differentiation into myofibroblasts, resulting in chronic allograft injury due to fibrosis [45,46]. MRAs, especially spironolactone, proved to be a useful tool in stabilizing myocardial fibrosis in mice with autoimmune myocarditis [47]. Recent studies have demonstrated that spironolactone, among its multiple pleiotropic effects, including the reduction of proinflammatory markers, may also have an influence on fluid–coagulant balance by regulating markers of hemostasis maintenance [48,49,50].

In a double-blind, randomized, placebo-controlled study, Morales-Buenrostro et al. showed that 100 mg of spironolactone 3 days before and 5 days after kidney transplantation reduces oxidative damage and tubular injury, which may lead to graft rejection [51]. However, controversially, in a double-blind, randomized, placebo-controlled trial that included 80 renal transplant patients, spironolactone did not improve markers of vascular inflammation and endothelial dysfunction [52]. Peled et al. found a significant correlation between the administration of spironolactone in heart transplantation in the preoperative period and a reduced risk of primary graft rejection, in addition to a decrease in in-hospital mortality, effects that are consistent even after 5 years post-transplant [53].

Furthermore, the blockade of aldosterone with spironolactone decreases inflammation, endothelial glycocalyx dysfunction, and neutrophil extracellular trap formation (NETosis), leading to diastolic cardiac function improvement [54]. NETs also showed potential involvement in AGR in lung, liver, and kidney transplantation [55,56,57].

**Figure 3:**
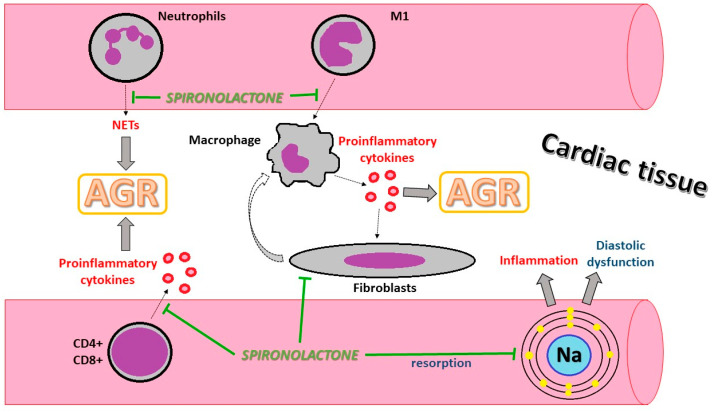
Illustration of the possible protective cellular mechanism of spironolactone in AGR. AGR: acute graft rejection; CD: clusters of differentiation; M1: monocyte type 1; Na: natrium; NETs: neutrophil extracellular traps.

The accumulation of sodium in the interstitial space is considered to be involved in tissue cytokine activation and inflammation (Figure 3). Moreover, diastolic dysfunction possesses prognostic value in patients with cardiac transplantation, with early diastolic dysfunction being a marker of a reduced rate of survival [58]. Furthermore, AGR might influence diastolic function as well as chronic graft rejection [59].

In selected end-stage HF patients with infiltrative, restrictive, and hypertrophic CM, heart transplantation is recommended [60], with no significant difference in survival rates amongst patients with restrictive CM compared to non-restrictive CM. However, patients with amyloidosis and cancer-therapy-related restrictive CM showed a decreased long-term survival rate [61]. On the other hand, in specific circumstances, patients are not suitable candidates for heart transplantation or MCS therapy. A total artificial heart might offer a solution, but the currently available data are insufficient [60]. Nevertheless, the Food and Drug Administration approved a total artificial heart with an indication in the bridge-to-transplant strategy, with a good one-year survival [62].

### 4.1. Future Perspectives in Cardiac Transplantation

The field of heart transplantation is continuously growing and improving since the first human-to-human transplant in 1967. The shortage of human organs is still a challenging medical deficiency, with no definitive solution to this day [63]. Xenograft heart transplantation from pigs might prove to be a promising strategy to overcome this challenge, primarily because of the favorable anatomical similarities. To date, there are two patients who benefited from xenograft transplantation at the University of Maryland, Baltimore. One of the individuals survived for a period of 2 months, but a combination of renal failure, antibody-mediated rejection, and porcine cytomegalovirus infection was registered as the cause of death [64,65]. The second patient lived 45 days, but complications such as renal failure, together with AGR, led to multiple organ dysfunction syndrome and death [66].

Potentially life-threatening complications that may occur in xenograft transplant that must be addressed are the numerous organs that contain porcine antigens and the risk of human infection by cross-species transmission [67]. Yang L et al. managed to inactivate and block the transmission of 62 copies of porcine endogenous retroviruses (PERVs) to human cells [68], thereby increasing optimism for future research despite many challenges that are yet to have been overcome.

Another promising strategy is out-of-body heart preservation. In the PROCEED II trial, which took place between 2010 and 2013, 130 patients were assigned either to the Organ Care System (the only clinical platform for the ex vivo perfusion of human donor hearts) or standard cold storage. At 30 days, the results for patient and graft survival rates as well as for cardiac serious adverse effects were comparable between groups. The authors also concluded comparable short-term clinical results [69].

Dhital et al. highlighted favorable results on cardiac transplantation with donors that underwent circulatory death for periods longer that two months, despite two patients needing temporary mechanical support [70]. These outcomes were reproduced by Messer et al. on nine patients using normothermic regional perfusion with no episodes of rejection [71].

One of the main causes of allograft loss is represented by cardiac allograft vasculopathy (CAV). Calcineurin inhibitors, a mammalian target of rapamycin inhibitors (mTORs), and purine synthesis inhibitors, pose effects on the function of the cardiovascular system [72,73]. It has been observed that mycophenolate mofetil can have the ability to delay the development and progression of CAV [74].

Moreover, dealing with CAV is challenging. Novel therapies such mTOR inhibitors showed to be non-inferior to classic immunosuppression therapies but with an increased risk of graft rejection [75]. Another component of the therapeutic arsenal is represented by monoclonal antibodies such as rituximab, basiliximab, bortezomib, and eculizumab, used in various stages after heart transplantation [67]. A promising therapy is targeting the inhibition of the CD28/B7 co-stimulatory pathway, inhibiting T cell activation, but without the rate of complications of classical immunosuppression [76]. Unfortunately, there is a therapeutic gap in CAV prevention, with no pharmaceutical product available to this day [77].

In terms of the prognosis for these patients, cell-free deoxyribonucleic acid (DNA) is released in circulation in small amounts through the process of cell death; this process was first discovered in 1948 [78]. In a cohort study including 171 patients with cardiac transplantation, donor-derived cell-free DNA led to the early detection of AGR with a 99% negative predictive value, reducing the need for endomyocardial biopsy by 81% of patients both for acute cellular and humoral rejections [79].

Heart transplant patients are exposed to a greater risk of developing HF with preserved ejection fraction (HFpEF) throughout their life following transplantation. Many risk factors, such obesity, diabetes, hypertension, graft rejection, and chronic vasculopathy, along with diastolic dysfunction, inflammation, microvascular disease, and fibrosis, are involved in this process as pathogenic mechanisms [80]. It has been observed that the majority of patients with graft rejection show no significant changes in left ventricle ejection fraction or standard global systolic functions [81]. The surgical technique may represent an affordable solution for preserving diastolic function. A cross-sectional study including 25 patients demonstrated that creating a smaller left atrium could enhance diastolic filling by assisting hydraulic forces and thereby improving the geometry between the left atrium and left ventricle [82].

SGLT2i showed overall safety and antihyperglycemic effects in renal and cardiac transplantation similar to the general population [83]. EMPA-Htx is an ongoing randomized controlled trial meant to assess the safety and efficacy of empagliflozin in cardiac transplant patients [84]. SGLT2i plays an important role in the modern treatment of HFpEF. The European Society of Cardiology (ESC) recommends SGLT2i as class I of indication in HFpEF based on the EMPEROR-Preserved and DELIVER trials. Both empagliflozin and dapagliflozin reduced HF hospitalizations in patients with and without T2DM. However, no significant reduction in cardiovascular death was seen [85,86,87]. Taking into consideration the benefit of SGLT2i in patients with HFpEF while also possessing cardiovascular and renal benefits, the administration of this drug in cardiac transplantation needs to be evaluated.

Moreover, in a recent meta-analysis that included patients with HF, sacubitril/valsartan showed better results in the composite outcome of hospitalizations for HF and cardiovascular death in HF patients, including individuals with HFpEF [88]. Basile C et al. previously studied the effect of sacubitril/valsartan in HFpEF, showing a reduction in composite HF decompensation along with all-cause mortality [89].

Taking into consideration MRAs, in the TOPCAT trial that included patients with HFpEF, the administration of spironolactone did not show benefits in death from cardiovascular causes or hospitalization for HF [90]. Nevertheless, in a subgroup analysis of patients from America, spironolactone use was associated with a reduced risk of cardiovascular death and all-cause mortality [91]. Additional benefits were seen in patients with resistant hypertension in a subgroup of the TOPCAT trial, with spironolactone reducing the risk even more in the primary outcomes [92]. In a previous study carried out at our institute, chronic treatment with spironolactone was associated with a lower risk of AGR in the first 2 months after cardiac transplantation [93].

Finally, in heart transplant patients, drug therapies that have shown beneficial effects in HFpEF need to be evaluated. Considering the risk of HFpEF development in this category of patients, alongside the serious adverse effects of the post-transplant immunosuppressive treatment, there might be advantages in using HFpEF drugs after transplantation.

### 4.2. Limitations

This research has a number of limitations that have to be mentioned. Firstly, the retrospective design imposes possible losses to follow-up, information bias, and the absence of data on potential confounding factors. Secondly, the relatively low number of patients from our cohort might result in false-positive or negative information, which can overestimate the associations between the drug therapy and the events by misinterpretation of CIs and statistical significance [94]. The reduced number of transplant patients comes from the lack of donor organs, which is caused by the lack of transplantation campaigns and legislation forcing the need to obtain an informed consent form signed by the donor’s family members a short time from the death of a close relative. Lastly, regarding the validation of our results, further prospective research with larger cohorts is vital in order to determine definitive conclusions.

## 5. Conclusions

In our study, early spironolactone initiation after heart transplant using a relatively low dosage represented a protective factor against 1-year and 2-year AGR but without a statistical benefit at 6 months. Moreover, spironolactone administration had a lower 2-year mortality rate. The protective role of spironolactone may be derived from its anti-inflammatory and immune-modifying properties, with suppressant effects of the proinflammatory cytokines that have been proven to have consequences on the occurrence of AGR and survival. Further prospective studies and larger cohorts are needed to validate our results.

## Figures and Tables

**Table 1:** Baseline characteristics.

	Total (n = 36)	Spironolactone (n = 14)	Non-Spironolactone (n = 22)	*p* Value
Daily dosage, mg (mean, SD)		33.3 (12.2)		
Age, years (mean, SD/median, 25th–75th IQR)	40,417 (13,561)/ 41,000 (39,000–47,341)	39,214 (17,290)/ 41,000 (24,957–54,104)	41,182 (10,949)/ 42,000 (39,000–47,047)	0.678 *
Females (n, %)	3 (8.3)	2 (14.3)	1 (4.5)	0.016 †
BMI, kg/m^2^ (mean, SD/median, 25th–75th IQR)	23,439 (4,574)/ 24,100 (22,661–25207)	23,086 (4.988)/ 23,900 (19,085–25,800)	23,664 (4.396)/ 24,100 (21,835–26,600)	0.717 *
BSA, m^2^ (mean, SD/median, 25th–75th IQR)	1.849 (0.319)/ 1.935 (1.749–1.993)	1.754 (0.381)/ 1.825 (1.604–1.982)	1.910 (0.265)/ 1.975 (1.730–2.031)	0.155 *
Ischemic CM (n, %)	8 (22.2)	4 (28.6)	4 (18.2)	0.075 †
Pre-T2DM (n, %)	3 (8.3)	3 (21.4)	0 (0.0)	0.061 †
Pre-BBs (n, %)	28 (77.8)	10 (71.4)	18 (81.8)	0.904 †
Pre-carvedilol (n, %)	25 (69.4)	9 (64.3)	16 (72.7)	0.560 †
Pre-ACEIs (n, %)	17 (47.2)	8 (57.1)	9 (40.9)	0.589 †
Pre-ramipril (n, %)	12 (33.3)	6 (42.9)	6 (27.3)	0.469 †
Pre-MRAs (n, %)	33 (91.7)	14 (100.0)	19 (86.4)	0.003 †
Pre-spironolactone (n, %)	29 (80.6)	13 (92.9)	16 (72.7)	0.469 †
Duration of inotropes/vasopressors, days (mean, SD/median, 25th–75th IQR)	6.806 (6.765) 4.500 (4.000–6.000)	4.857 (4.036)/ 4.000 (3.000–5.104)	8.045 (7.877)/ 5.000 (4.000–7.000)	0.081 **
ICU stay, days (mean, SD/median, 25th–75th IQR)	53,056 (68,201) 34,000 (30,659–39,341)	35,286 (18,370)/ 32,500 (28,000–35,104)	64,364 (84,847)/ 38,500 (30,953–42,935)	0.163 **
Hospital stay, days (mean, SD/median, 25th–75th IQR)	61,833 (71,952)/ 38,500 (34,000–46,411)	41,286 (19,948)/ 37,000 (33,583–53,834)	74,909 (89,003)/ 42,000 (33,906–64,093)	0.236 **
2R/3R AGR (n, %)	3 (8.3)	1 (7.1)	2 (9.1)	0.084 †
6-month mortality (n, %)	4 (11.1)	1 (7.1)	3 (13.6)	0.084 †
2-year mortality (n, %)	4 (11.1)	1 (7.1)	3 (13.6)	0.084 †

* Student’s *t* test; ** Mann–Whitney test; † Chi-square test.

**Table 2:** Early treatment with spironolactone in 6-month, 1-year, and 2-year AGR.

	Spironolactone RR Value (95%CI)	NNT (95%CI)	*p* Value
6-month AGR	0.286 (0.074–1.101)	2.800 (1.522–17.491)	0.068
1-year AGR	0.262 (0.069–0.999)	2.484 (1.425–9.655)	0.049
2-year AGR	0.337 (0.118–0.964)	2.369 (1.376–8.526)	0.042

**Table 3:** Details regarding the first documented AGR episodes.

Early Spironolactone Usage	AGR Grade	Duration to First AGR (Months)	AGR Type
With spironolactone	1R	1	ACR
	2R	13	ACR
	1	2	AMR
Without spironolactone	1	1	AMR
	1	18	AMR
	2	23	AMR
	1R	2	ACR
	1	2	AMR
	1	3	AMR
	1R	11	ACR
	1	2	AMR
	1R	3	ACR
	1R	1	ACR
	1R	5	ACR
	1	1	AMR
	1R	2	ACR
	3	2	AMR

## Data Availability

The data that support the findings of this study are available from the corresponding author upon reasonable request.
